# Tailoring Al_2_Y Reinforcement in Mg–Al–Ca–Mn Composites via a Master Alloy Approach: Microstructure Evolution and Strength–Ductility Synergy

**DOI:** 10.3390/ma19194180

**Published:** 2026-09-30

**Authors:** Jiang Zhang, Zhongtao Jiang, Yifeng Zou, Shan Wan, Ming Wang

**Affiliations:** 1 College of Materials Science and Engineering, Guilin University of Technology, Guilin 541004, China; zhangjiang@cmhk.com; 2 College of Materials Science and Engineering, Chongqing University of Arts and Sciences, Chongqing 402160, Chinaeuynahs1028@163.com (S.W.)

**Keywords:** magnesium matrix composite, Al_2_Y reinforcement, master alloy, dynamic recrystallization, bimodal microstructure, tensile properties

## Abstract

**Highlights:**

**What are the main findings?**

- A Mg–12Al_2_Y master alloy controlled Al_2_Y reinforcement in Mg–Al–Ca–Mn composites.
- The 1 wt.% Al_2_Y composite reached the highest yield strength of 346 MPa.
- The 3 wt.% Al_2_Y composite combined 316 MPa yield strength with 11.8% elongation.

**What are the implications of the main findings?**

- Al_2_Y content governs particle dispersion, recrystallization, texture, and retained strain.
- Moderate Al_2_Y addition gives a better strength–ductility balance than excess addition.
- Master-alloy addition offers a practical route to tailor low-alloy Mg composites.

**Abstract:**

Balancing strength and ductility remains a central challenge in low-alloy Mg–Al–Ca–Mn alloys. In this study, a Mg–12Al_2_Y master alloy was added to Mg–1.2Al–0.8Ca–0.4Mn to obtain nominal Al_2_Y contents of 0, 1, 3, and 5 wt.%. The composites were prepared by stir casting and hot extrusion. In the as-cast condition, Al_2_Y particles occurred both within α-Mg grains and in interdendritic regions; the 3 wt.% Al_2_Y composite had the finest structure. Extrusion produced a bimodal microstructure of fine dynamically recrystallized (DRXed) grains and coarse unrecrystallized regions, and fragmented Al_2_Y and Al_2_Ca particles formed stringers along the extrusion direction. The 1 wt.% Al_2_Y composite showed the highest tensile yield strength (346 MPa) and ultimate tensile strength (361 MPa), with an elongation of 5.8%. At 3 wt.%, the yield strength, ultimate tensile strength, and elongation were 316 MPa, 332 MPa, and 11.8%, respectively, representing the best strength–ductility balance among the compositions examined. Increasing the Al_2_Y content to 5 wt.% raised the elongation to 12.6% but reduced the strength because of particle agglomeration and microstructural coarsening. The variation in mechanical response is discussed in relation to recrystallized grain size, retained deformation, texture, and particle distribution. Within the processing window examined here, master-alloy addition offers a practical route for controlling Al_2_Y reinforcement in Mg–Al–Ca–Mn composites.

## 1. Introduction

Magnesium alloys combine low density with high specific strength and are therefore attractive for lightweight structural components [1,2,3]. Their broader application, however, is constrained by low stiffness, moderate strength, and limited formability at room temperature. Alloying and microstructural control can improve mechanical performance while preserving the weight advantage of Mg. In Mg–Al alloys, rare-earth additions alter the morphology of Mg_17_Al_12_ and promote thermally stable intermetallic phases [4,5,6,7]. Calcium provides a less expensive option: secondary phases formed during solidification refine the microstructure and can improve tensile properties [8,9].

The mechanical response of Mg–Al–Ca alloys is governed in large part by the type, size, and distribution of their secondary phases. For example, rolled Mg–Al–Ca–Zn–Mn alloys containing dense nanoscale Mg_17_Al_12_ precipitates retain appreciable elongation at high ultimate strength [10]. In extruded Mg–6Al–1Ca, deformable Al_2_Ca and dislocation accumulation around Al_2_Ca precipitates enhanced work hardening and yielded an elongation of 27% [11]. Thus, suitably controlled secondary phases can provide strengthening without necessarily suppressing strain accommodation.

Introducing reinforcing particles is another way to strengthen Mg matrices [12,13,14]. Zhang et al. [15] found that 1 vol.% SiC provided nucleation sites for Al_2_Ca in a Mg–Al–Ca composite, refining the phase and restricting grain growth. The accompanying loss of elongation also illustrates the penalties associated with interfacial reactions, chemical incompatibility, and thermal-expansion mismatch [16]. Al_2_Y is of interest because it is stable and forms a favorable interface with Mg. In AZ91–1Y, coarse Al_2_Y particles together with submicrometer Mg_17_Al_12_ precipitates controlled a bimodal grain structure and improved the strength–ductility balance [17]. In rolled AZ31, Al_2_Y promoted particle-stimulated nucleation (PSN), grain refinement, and changes in rolling texture [18].

Previous work has established that Al_2_Y affects recrystallization, texture, and mechanical behavior in Mg–Al alloys, but its concentration-dependent effects in a low-alloy Mg–Al–Ca–Mn matrix have not been resolved. Here, Mg–1.2Al–0.8Ca–0.4Mn composites with 0, 1, 3, and 5 wt.% nominal Al_2_Y were gravity-cast using a Mg–12Al_2_Y master alloy and then hot-extruded. The as-cast and extruded microstructures were related to room-temperature tensile properties. Particular attention was given to the strength maximum at 1 wt.% Al_2_Y, the improved strength–ductility balance at 3 wt.%, and the decline in strength at 5 wt.%.

## 2. Materials and Methods

Table 1 lists the nominal compositions of the Mg–Al–Ca–Mn composites. The designation AXMW1208X uses X (0, 1, 3, or 5) to indicate the nominal Al_2_Y content in wt.%. Inductively coupled plasma atomic-emission spectroscopy (ICP-AES; Avio 500, PerkinElmer Management (Shanghai) Co., Ltd., Shanghai, China) gave measured compositions of Mg–1.18Al–0.75Ca–0.43Mn, Mg–1.60Al–0.77Ca–0.38Mn–0.61Y, Mg–2.37Al–0.81Ca–0.40Mn–1.8Y, and Mg–3.20Al–0.78Ca–0.37Mn–3.15Y.

### 2.1. Composite Preparation

The as-cast composites were prepared in an electric resistance furnace (VBF-1200X-H, Hefei Ke Jing Materials Technology Co., Ltd. (Hefei, China) at 720 °C. The charge consisted ofpure Mg (>99.9 wt.%), pure Al (>99.9 wt.%), Mg—18 wt.% Ca, Mg—3.5 wt.% Mn, and Mg—12 wt.% Al_2_Y master alloys (Ganzhou FeiTeng Light Alloy Co., Ltd. (Ganzhou, China). The Al_2_Y particles were synthesized as described previously [19] and introduced through the Mg—12 wt.% Al_2_Y master alloy. Stir casting was performed in a stainless-steel crucible (Shenzhen Zhijia Instrument Equipment Co., Ltd., Shenzhen, China) under a protective atmosphere of 99 vol.% CO_2_ and 1 vol.% SF_6_. After refining and slag removal, the melt was held at 720 °C for 20 min and poured into a cylindrical permanent mold (Foshan Nanhai Platinum Strong Machinery Equipment Co., Ltd., Foshan, China) preheated to 250 °C.

### 2.2. Homogenization and Hot Extrusion

The composite ingots were homogenized in two steps, first at 450 °C for 8 h and then at 500 °C for 4 h, followed by water quenching. Cylindrical billets (Φ30 mm × 34 mm) were machined from the homogenized ingots and preheated at 300 °C for 30 min. Hot extrusion was conducted at 300 °C with a ram speed of 2 mm/s and an extrusion ratio of 7:1. The extruded rods (Φ11 mm) were immediately quenched in water.

### 2.3. Microstructural Characterization

Specimens for microstructural characterization were taken from the central region of the extruded rods. Optical microscopy (OM) specimens were mechanically ground, polished, etched with an acetic–picric solution (Runyou Chemical (Shenzhen) Co., Ltd. (Shenzhen, China), and examined using an LSXTL18A microscope(Shanghai Dilun Optical Instrument Co., Ltd. Shanghai, China). Further characterization used a field-emission scanning electron microscope (SEM) (GeminiSEM 300, (Carl Zeiss Microscopy GmbH, Jena, Germany)) equipped with energy-dispersive X-ray spectroscopy (EDS) (Oxford Instruments NanoAnalysis, High Wycombe, UK). Electron backscatter diffraction Electron backscatter diffraction (EBSD) was performed using a Symmetry detector (Oxford Instruments NanoAnalysis, High Wycombe, UK) at an accelerating voltage of 20 kV and a step size of 0.12 μm. Kernel average misorientation (KAM) was calculated using first-nearest-neighbor points with a 5° cutoff angle. The KAM data were used to compare local lattice distortion and the relative geometrically necessary dislocation (GND) density among specimens. SEM specimens were etched in 4 vol.% nitric acid ((Runyou Chemical (Shenzhen) Co., Ltd. Shenzhen, China) and 96 vol.% ethanol (Runyou Chemical (Shenzhen) Co., Ltd. Shenzhen, China). EBSD specimens were electropolished in ACII solution (Runyou Chemical (Shenzhen) Co., Ltd. Shenzhen, China) at −20 °C for 90 s at 20 V DC. Transmission electron microscopy (TEM) was conducted using an FEI Tecnai G2 F20 microscope (Thermo Fisher (Shanghai) Scientific, Shanghai, China) operated at 200 kV. X-ray diffraction (XRD) patterns were collected using a PANalytical Empyrean 03030502 diffractometer (Malvern Panalytical Instruments, China, Shanghai, China) over 2θ = 15–90° at 45 kV and 40 mA with a step size of 0.01°.

### 2.4. Mechanical Testing

Room-temperature tensile tests were conducted on the as-extruded rods using a WD-200 electronic universal testing machine (Shandong Landing Photoelectricity Technology Co., Ltd. (Weifang, China) at a crosshead speed of 1 mm min^−1^. Dog-bone specimens had a gauge length of 34 mm, a width of 3 mm, and a thickness of 1.9 mm. Three specimens were tested for each composition.

## 3. Results

### 3.1. Microstructure of the As-Cast Composites

**Figure 1:**
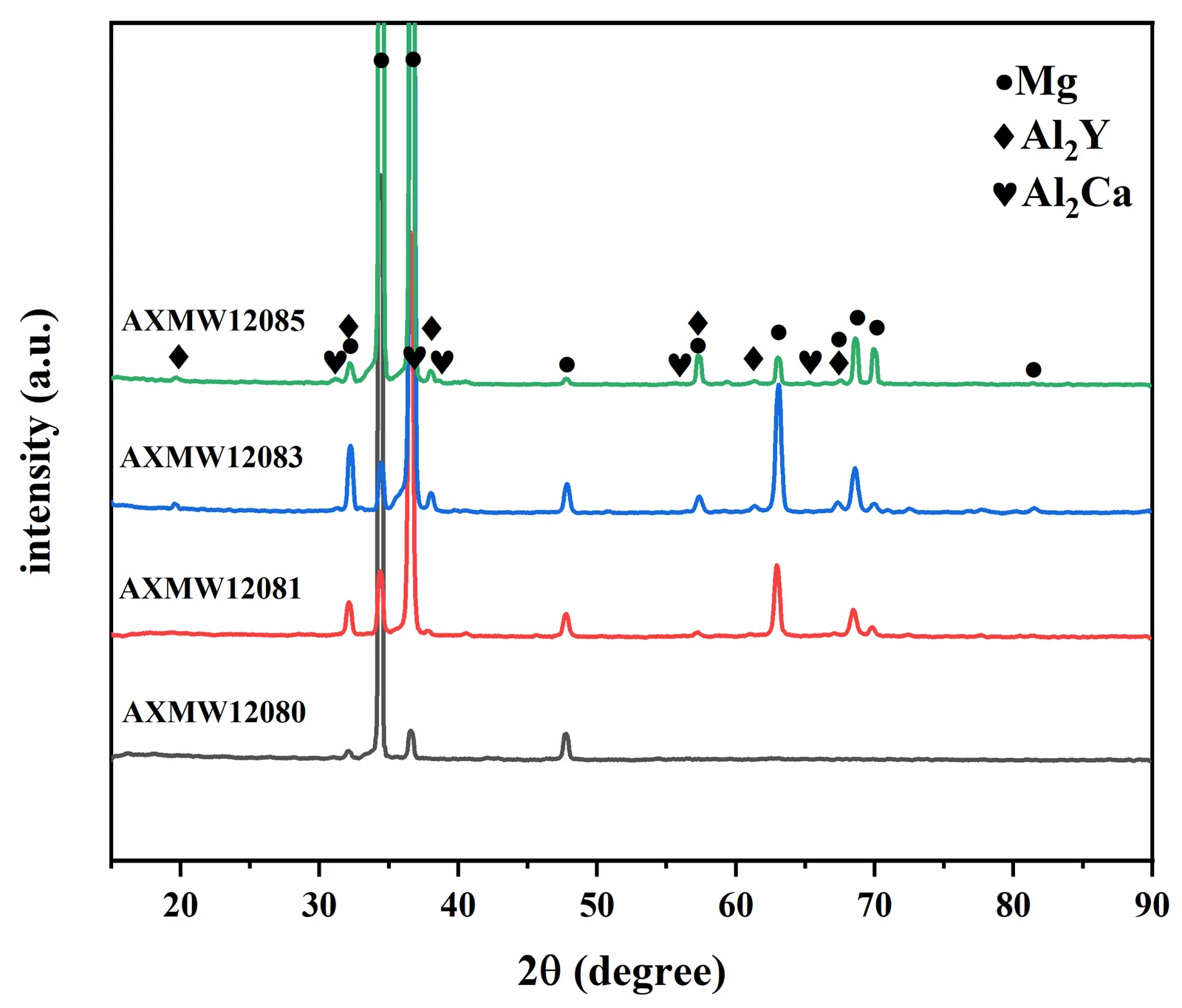
XRD patterns of the as-cast AXMW1208X composites.

The X-ray diffraction (XRD) patterns of the as-cast composites are presented in Figure 1. AXMW12080 consisted predominantly of α-Mg, with weak peaks assigned to cubic C15 Al_2_Ca. No β-Mg_17_Al_12_ peak was resolved, which is compatible with preferential formation of Al_2_Ca in the Ca-containing matrix. Characteristic Al_2_Y peaks appeared in AXMW12081, AXMW12083, and AXMW12085 after addition of the Mg–12Al_2_Y master alloy, and their relative intensities increased with nominal Al_2_Y content. No other principal matrix phase was detected.

**Figure 2:**
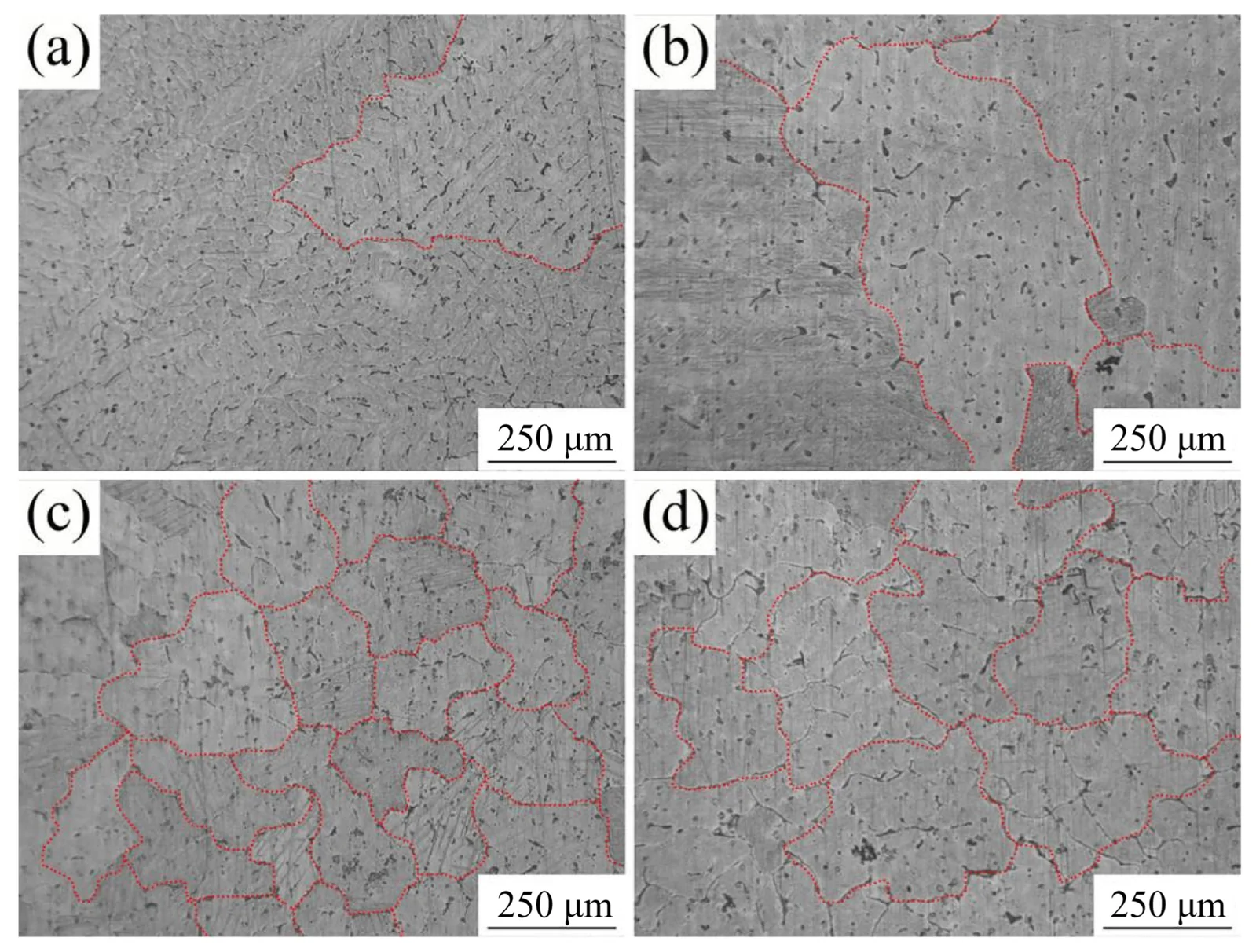
Optical micrographs of the as-cast AXMW1208X composites: (**a**) AXMW12080; (**b**) AXMW12081; (**c**) AXMW12083; (**d**) AXMW12085. The red dashed lines indicate the grain boundaries.

The as-cast optical microstructures (Figure 2) contained primary α-Mg dendrites and interdendritic secondary phases in every composition. AXMW12083 had the finest dendritic structure and a relatively uniform distribution of secondary phases. At 5 wt.% Al_2_Y, the particles were coarser and more strongly clustered, and refinement was less pronounced. Al_2_Y can act as a heterogeneous nucleation substrate for α-Mg [20]; particle coarsening and clustering would reduce the effective number density of such dispersed sites.

**Figure 3:**
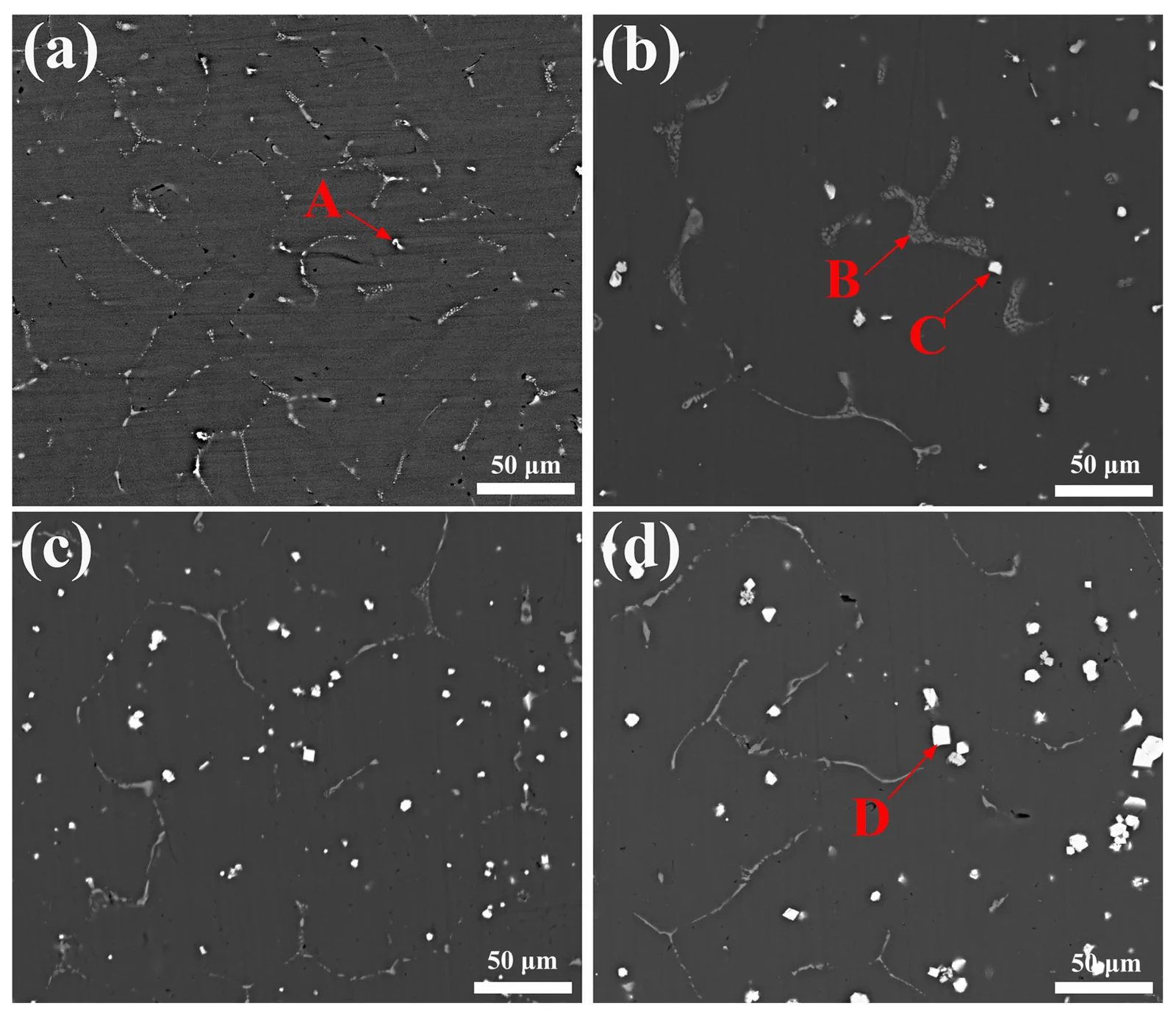
SEM images of the as-cast AXMW1208X composites: (**a**) AXMW12080; (**b**) AXMW12081; (**c**) AXMW12083; (**d**) AXMW12085. The red labels A–D indicate the locations selected for EDS point analyses.

Figure 3 presents the morphology and distribution of the secondary phases in the as-cast composites, and the corresponding EDS results are summarized in Table 2. In AXMW12080, the combined XRD and EDS evidence identifies the interdendritic phase as Al_2_Ca. Points A and B had Al/Ca atomic ratios close to 2. The granular, filamentary, and lamellar morphologies within interdendritic channels are characteristic of a constituent formed from solute-enriched residual liquid.

Addition of the Mg–12Al_2_Y master alloy introduced a second phase. At points C and D, the Al/Y atomic ratio was approximately 2, identifying this phase as Al_2_Y. Although Al_2_Y and Al_2_Ca both have the cubic C15 Laves structure, their morphologies were distinct. Al_2_Y was mainly present as faceted polygonal particles, with occasional discontinuous network-like features near dendrite boundaries. Al_2_Ca formed in situ during the final stage of solidification and remained concentrated in interdendritic channels. Al_2_Y, by contrast, entered with the master alloy and retained the faceted morphology of primary Al_2_Y crystallites; particles were consequently also found inside α-Mg grains.

The EDS signal at point D should be interpreted cautiously: the probe overlapped the small Al_2_Y particle and the surrounding α-Mg matrix, so the measured composition contains a substantial matrix contribution and does not represent a reliable stoichiometric analysis of Al_2_Y. This limitation has been added to avoid overinterpreting the D-point data.

The Al_2_Y particle population evolved with nominal content. At 1 wt.%, relatively fine particles were dispersed mainly within α-Mg grains. Raising the content to 3 wt.% increased the number density without substantially degrading the dispersion. At 5 wt.%, particles coarsened and agglomerated, particularly in interdendritic regions. Among the casting conditions studied, 3 wt.% produced the most favorable combination of particle size, number density, and dispersion.

### 3.2. Microstructure of the As-Extruded Composites

**Figure 4:**
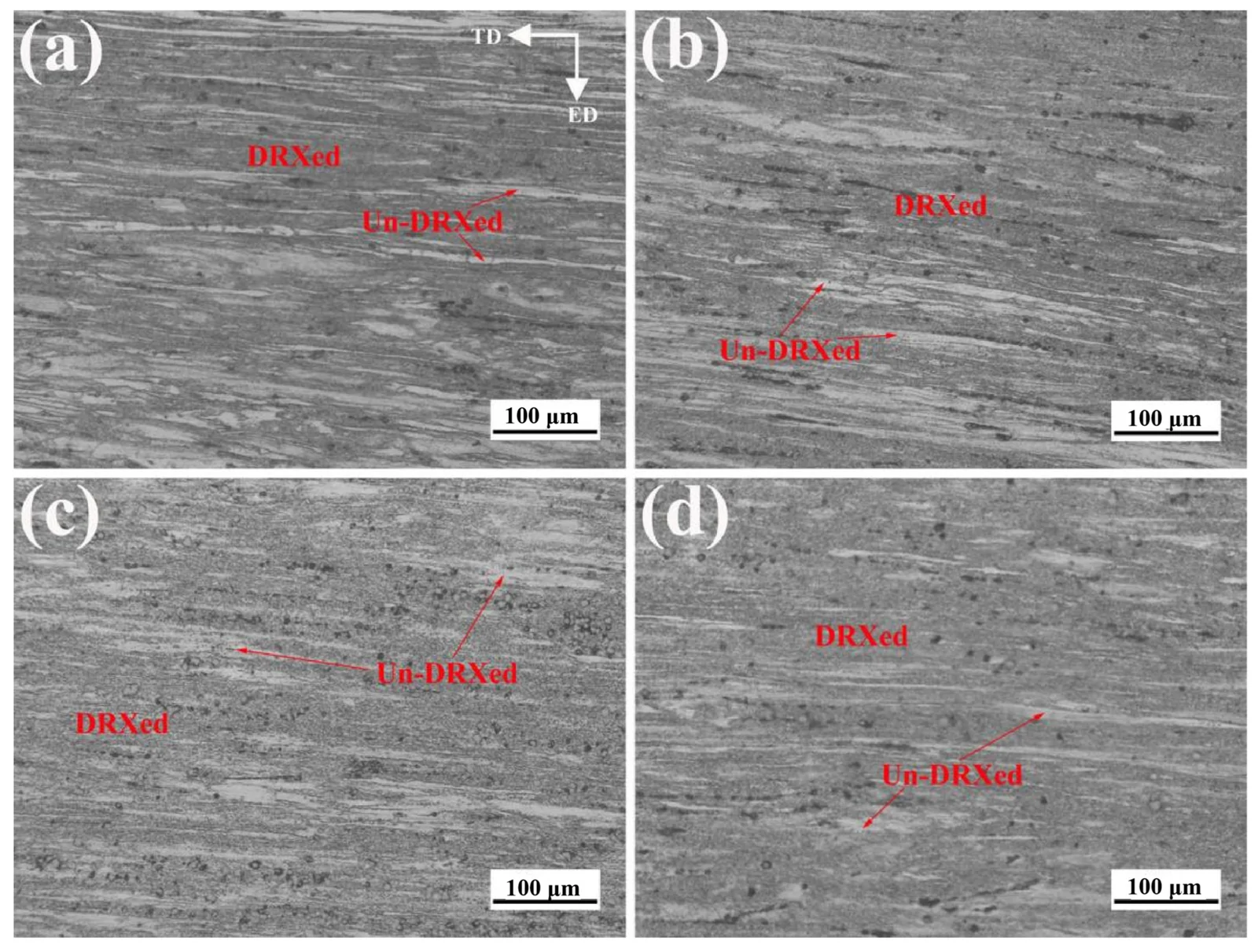
Optical micrographs of the as-extruded AXMW1208X composites in the longitudinal section: (**a**) AXMW12080; (**b**) AXMW12081; (**c**) AXMW12083; (**d**) AXMW12085. ED and TD denote the extrusion direction and transverse direction, respectively.

Longitudinal optical micrographs of the extruded composites are shown in Figure 4. Extrusion refined the grains and broke the secondary phases into stringers parallel to the extrusion direction (ED). All four compositions developed a bimodal structure containing fine dynamically recrystallized (DRXed) grains and coarse unrecrystallized regions [21,22].

The inhomogeneous post-extrusion structure originated from the coupled effects of the cast-state phase distribution and the heterogeneous strain imposed during extrusion. The interdendritic Al_2_Ca networks and introduced Al_2_Y particles fragmented into aligned stringers, while the particle-rich regions generated local strain gradients and particle-stimulated nucleation. Consequently, dynamic recrystallization proceeded more readily in some regions than in others, leaving fine DRXed grains adjacent to coarse unrecrystallized areas. The increasing particle population with Al_2_Y addition intensified this spatial heterogeneity, whereas particle coarsening and agglomeration at 5 wt.% reduced the effectiveness of refinement.

Dark bands parallel to the ED were present in all four composites. They originated from interdendritic networks that fragmented and redistributed during extrusion [12], although individual phases cannot be distinguished at this magnification. The bands were sparse in AXMW12080 and became more numerous after Al_2_Y addition as the total secondary-phase population increased.

The micrographs further indicate that Al_2_Y changed the relative proportions of DRXed and unrecrystallized material; AXMW12085 had the smallest apparent fraction of coarse unrecrystallized regions. Grain size and boundary character were quantified by EBSD.

**Figure 5:**
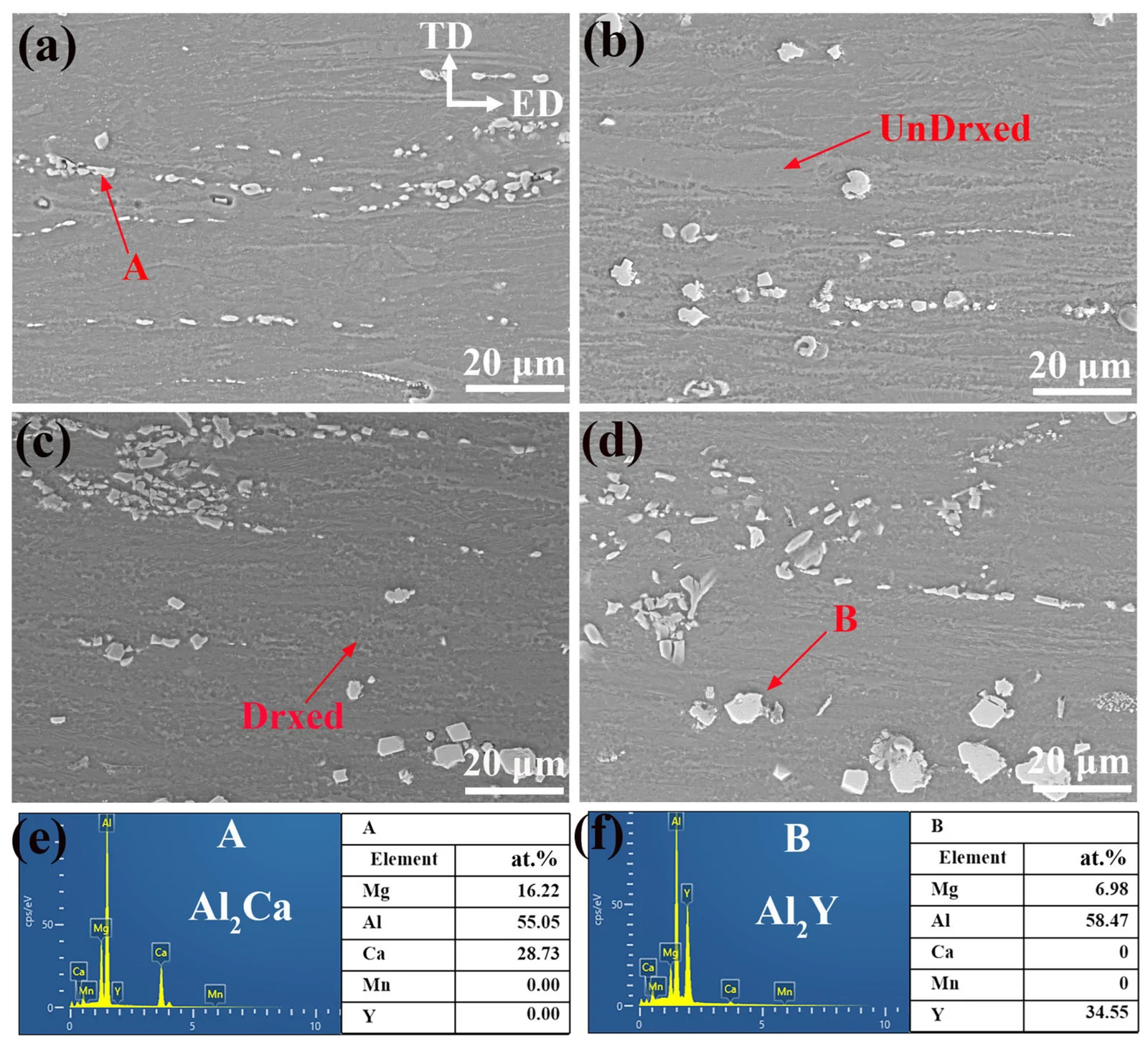
SEM images of the as-extruded AXMW1208X composites: (**a**) AXMW12080; (**b**) AXMW12081; (**c**) AXMW12083; (**d**) AXMW12085; (**e**) EDS results acquired at point A; (**f**) EDS results acquired at point B. The red labels A, B indicate the locations selected for EDS point analyses. ED and TD denote the extrusion direction and transverse direction, respectively.

Figure 5 shows the secondary phases after extrusion, with the ED horizontal. In AXMW12080 (Figure 5a), fragmented particles were dispersed through the matrix and aligned with the ED. A representative particle at point A (Figure 5e) contained mainly Al and Ca at an atomic ratio of about 2:1. In conjunction with the XRD results, this identifies the particle as Al_2_Ca. The phase persisted at 300 °C but fragmented under the applied strain. Fine equiaxed grains were commonly found next to the fragments.

The Al_2_Y-containing composites also contained bright polygonal particles several μm across (Figure 5b–d). The Al/Y atomic ratio at point B (Figure 5f) was approximately 2:1; the EDS composition, particle morphology, and XRD patterns identify these particles as Al_2_Y.

Fine equiaxed grains surrounded both fragmented Al_2_Ca and polygonal Al_2_Y particles in all four composites, and deformation zones were visible beside the particles. Particle size is known to affect dynamic recrystallization in Mg-matrix composites [23,24], making these local features compatible with PSN during extrusion. The images do not, however, separate the contributions of Al_2_Ca and Al_2_Y to the overall DRXed fraction.

**Figure 6:**
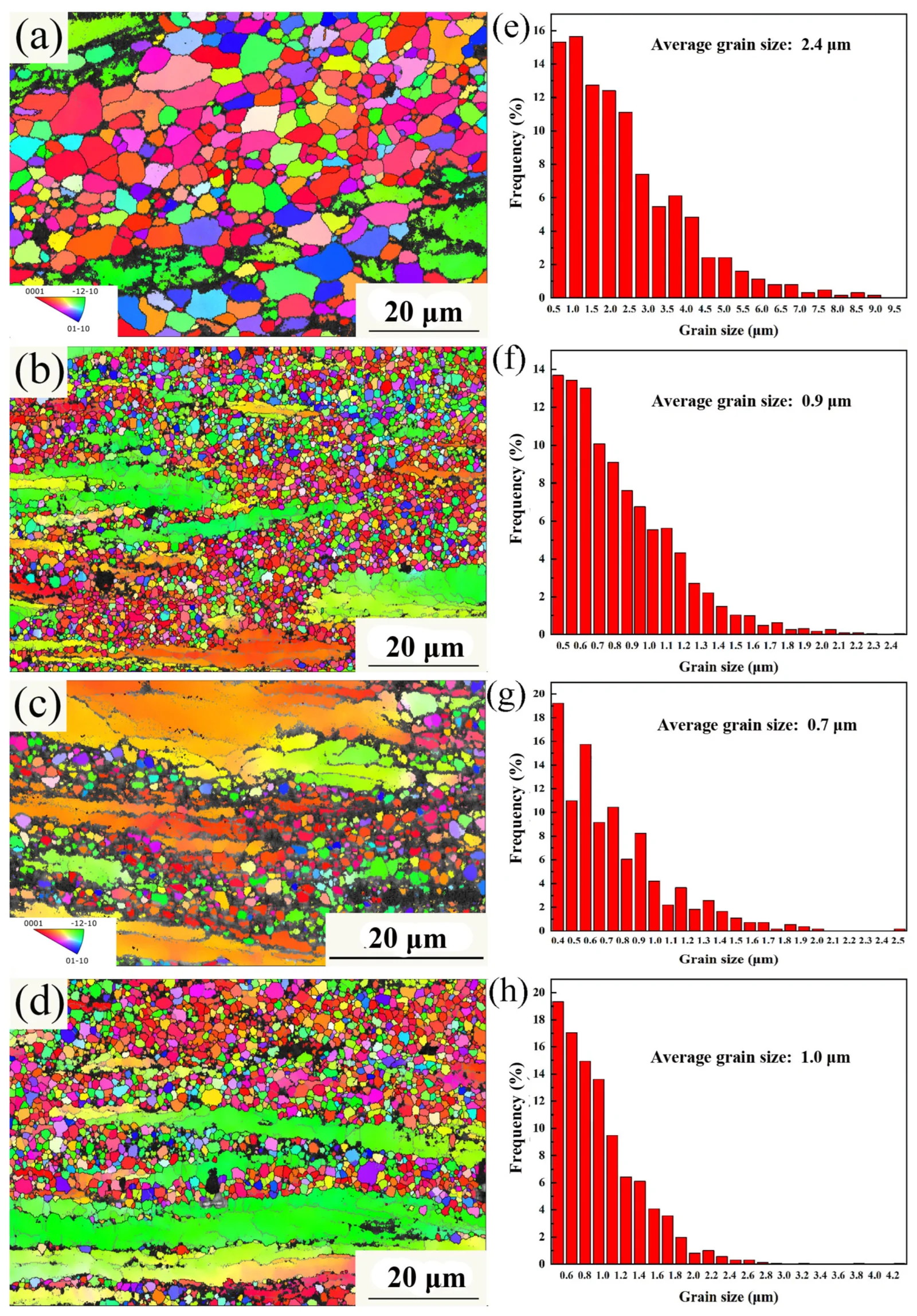
Inverse pole figure (IPF) maps and dynamically recrystallized (DRXed) grain size distributions of the as-extruded AXMW1208X composites: (**a**,**e**) AXMW12080; (**b**,**f**) AXMW12081; (**c**,**g**) AXMW12083; (**d**,**h**) AXMW12085.

Figure 6a–d present inverse pole figure (IPF) maps of the extruded composites with the ED horizontal; the corresponding DRXed grain-size distributions are given in Figure 6e–h. Each composite contained fine equiaxed DRXed grains and coarse elongated unrecrystallized grains. Low-angle grain boundaries (LAGBs, 2–15°) are marked in white and high-angle grain boundaries (HAGBs, >15°) in black. LAGBs occurred primarily within the elongated unrecrystallized grains, which retained a predominant <01-10>//ED orientation.

Both LAGB density and mean DRXed grain size varied nonmonotonically with Al_2_Y content. The mean DRXed grain size fell from 2.4 μm in AXMW12080 to 0.7 μm in AXMW12083, then increased at 5 wt.% Al_2_Y (Figure 6e–h). Thus, the finest DRXed structure occurred at 3 wt.% rather than at the highest particle content.

The phase content also altered the balance between recrystallized and unrecrystallized material. The unrecrystallized area fraction first increased and then decreased, while the DRXed fraction changed in the opposite direction. In each case, fine DRXed grains coexisted with coarse regions that retained the deformation structure [25,26].

**Figure 7:**
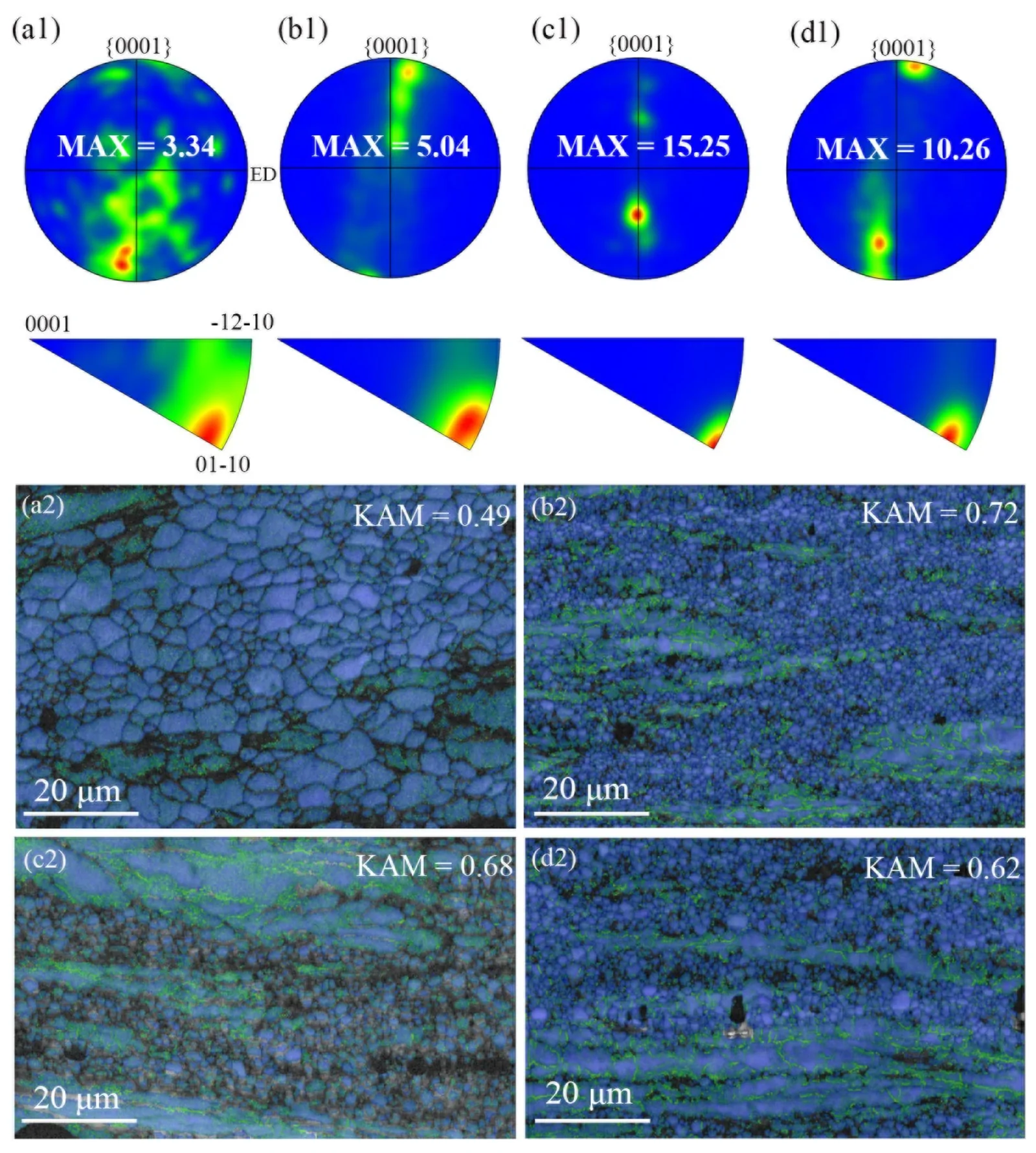
EBSD analyses of the as-extruded AXMW1208X composites: (**a1**,**a2**) AXMW12080; (**b1**,**b2**) AXMW12081; (**c1**,**c2**) AXMW12083; (**d1**,**d2**) AXMW12085. (**a1**–**d1**) Inverse pole figure (IPF) and pole figures (PF). (**a2**–**d2**) Kernel average misorientation maps and the KAM values.

The inverse pole figure (IPF) and pole figures (PF) in Figure 7(a1–d1) reveal an extrusion-fiber texture in all four composites, with the {0001} basal planes and <10-10> direction of α-Mg aligned with the ED. Basal texture intensity rose with Al_2_Y content. Likewise, the IPFs in Figure 7(a1–d1) show a progressively stronger <10-10>//ED component and greater intensity near the prismatic orientation. Al_2_Y addition therefore affected both recrystallization and the retained extrusion texture.

For loading parallel to the ED, the stronger basal texture is likely to influence the tensile response. It cannot by itself explain the strength trend, because strength peaked at 1 wt.% Al_2_Y rather than at the composition with the strongest texture. Texture is known to influence room-temperature plastic deformation in Mg alloys [27,28]. It is therefore treated here as one contribution to strength, alongside the other microstructural changes, rather than as a stand-alone explanation for the maximum. In the KAM maps (Figure 7(a2–d2), the highest local values occur within coarse unrecrystallized grains, indicating greater lattice curvature and a higher relative density of geometrically necessary dislocations (GNDs). Fine DRXed grains have lower KAM values because dynamic recrystallization removes part of the deformation-induced dislocation structure. Mean KAM values for AXMW12080, AXMW12081, AXMW12083, and AXMW12085 were 0.49°, 0.72°, 0.68°, and 0.62°, respectively. The maximum at 1 wt.% Al_2_Y and subsequent decline indicate a corresponding nonmonotonic variation in the relative retained GND density [29].

The relative GND density was estimated from the KAM data using Equation (1) [30].

(1) $$\rho_{\mathrm{GND}}=\frac{2{\bar{\theta }}_{\mathrm{KAM}}}{\mathrm{xb}}$$

In Equation (1), *ρ_GND_* denotes the estimated GND density, *θ_KAM_* the mean local misorientation in radians, *x* the effective kernel distance, and *b* the Burgers vector magnitude. For the first-nearest-neighbor calculation, *x* was taken as twice the EBSD step size (240 nm), and b was set to 0.32 nm for Mg-based systems. The resulting relative GND densities were 2.23 × 10^14^ m^−2^ for AXMW12080, 3.27 × 10^14^ m^−2^ for AXMW12081, 3.09 × 10^14^ m^−2^ for AXMW12083, and 2.82 × 10^14^ m^−2^ for AXMW12085. The highest value in AXMW12081 agrees with its mean KAM of 0.72°. Because the estimate depends on the KAM neighborhood, angular conversion, and length convention, these values are used only to compare specimens acquired under identical conditions.

**Figure 8:**
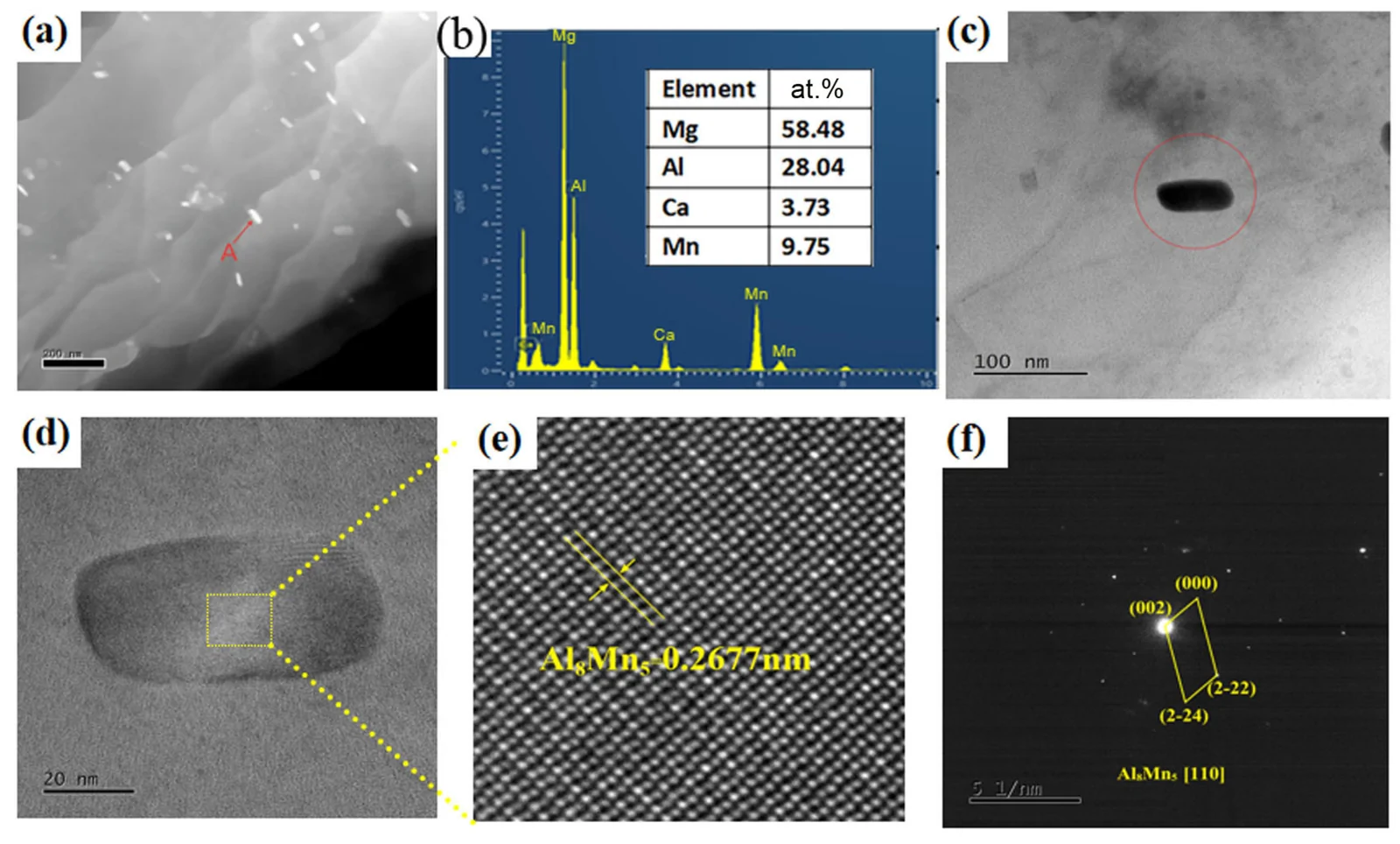
(**a**) Bright-field TEM image of the as-extruded AXMW12083 composite; (**b**) EDS result at point A; (**c**) magnified bright-field TEM image showing fine particles; (**d**,**e**) HRTEM image; (**f**) corresponding SAED pattern. The red circles indicate the precipitated phases.

The bright-field TEM image of AXMW12083 (Figure 8a) reveals numerous rod-shaped particles in the α-Mg matrix. TEM-EDS detected mainly Al and Mn. Preferential formation of an Al–Mn compound is thermodynamically plausible given the strongly negative mixing enthalpy of this pair [31].

High-resolution TEM (HRTEM) and selected-area electron diffraction (SAED) were used to identify the rod-shaped particles. Figure 8c shows the particles at higher magnification, and lattice fringes are resolved in Figure 8d,e. Indexing the SAED pattern in Figure 8f identified the phase as Al_8_Mn_5_.

Al_8_Mn_5_ has been reported to strengthen Mg alloys and to provide heterogeneous nucleation sites for α-Mg in Mg–Al-based systems [32,33,34]. In AXMW12083, it may therefore assist grain refinement and add a dispersion-strengthening contribution.

### 3.3. Mechanical Properties of the Composites

**Figure 9:**
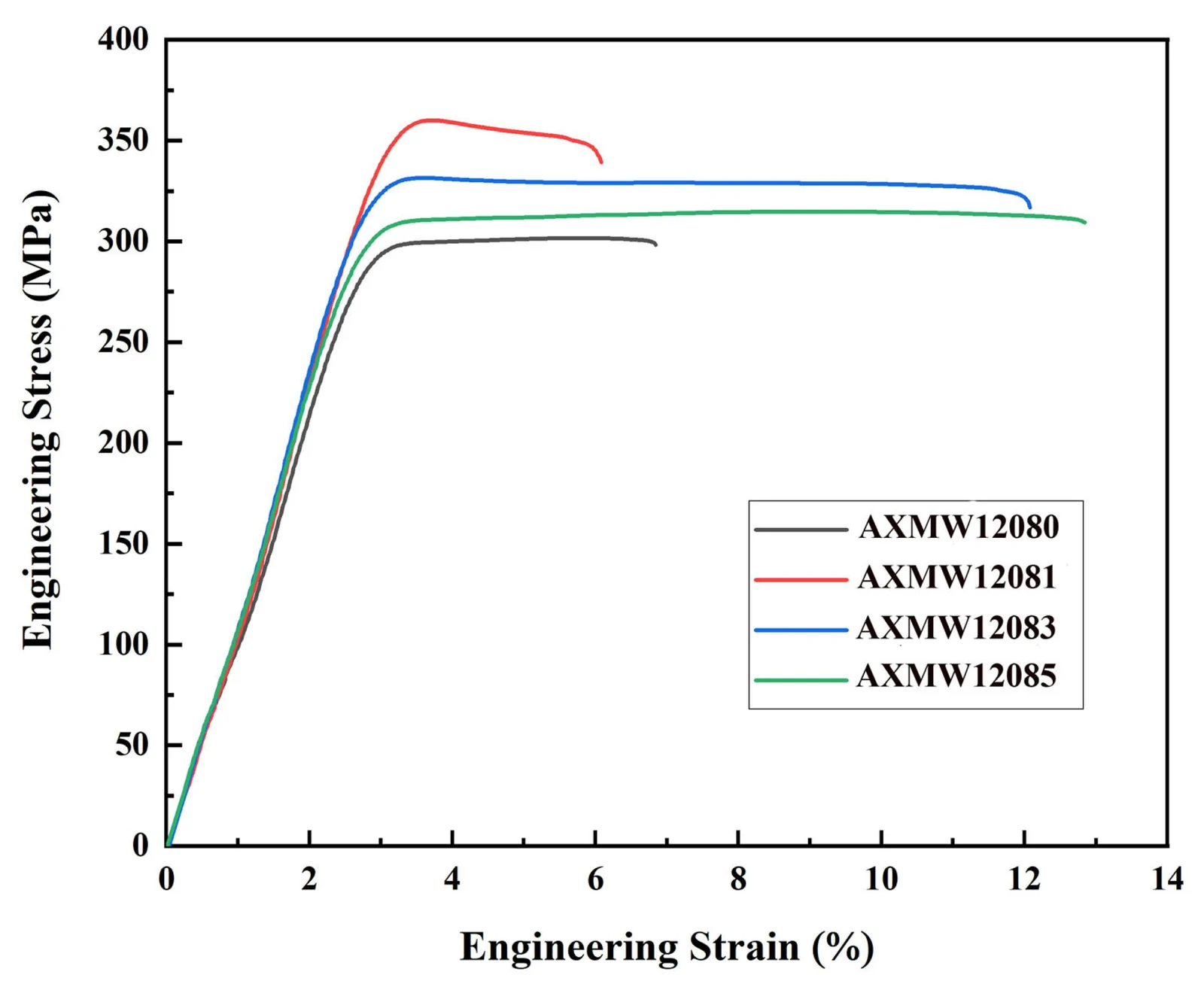
Tensile stress–strain curves of the as-extruded AXMW1208X composites.

The room-temperature engineering stress–strain curves are plotted in Figure 9, and Table 3 lists the tensile properties. Strength rose sharply at 1 wt.% Al_2_Y and declined with further additions. AXMW12081 reached a tensile yield strength (TYS) of 346 MPa and an ultimate tensile strength (UTS) of 361 MPa, compared with 285 and 300 MPa for AXMW12080, while elongation fell from 6.5% to 5.8%. AXMW12083 retained TYS and UTS values of 316 and 332 MPa and reached 11.8% elongation. AXMW12085 had the greatest elongation (12.6%) but lower TYS and UTS values of 296 and 315 MPa. On this basis, AXMW12083 offered the best strength–elongation balance among the compositions tested.

## 4. Discussion

### 4.1. Origin of the Composition-Dependent Strength

For loading parallel to the extrusion direction, the extrusion-fibre texture and its increasing intensity with Al_2_Y content can influence the relative activation of basal and non-basal deformation modes and therefore contribute to both the yield response and strain accommodation. However, texture alone cannot explain the strength maximum at 1 wt.% Al_2_Y, because the strongest texture occurred at a different composition. Its contribution must therefore be considered together with the grain structure, retained deformation, and particle distribution.

Grain size, secondary phases, retained deformation, and texture all contributed to the tensile response. The Hall–Petch relationship provides the conventional description of grain-boundary strengthening [35,36], while its slope in Mg alloys depends strongly on texture and deformation mode [37].

*σ_y_* = *σ*_0_ + *kd*^−1/2^ (2)

where, *σ_y_* is the yield stress, *σ*_0_ is the friction stress when the dislocation slides on the slip plane, *k* is the Hall-Page slope, and d is the average grain size. The Hall–Petch relation is useful qualitatively, but its contribution cannot be isolated from the present data because both the DRXed fraction and the grain-size distribution vary across the bimodal microstructures. In particular, AXMW12083 had the smallest DRXed grains but did not show the highest strength; grain refinement was therefore not the controlling factor on its own.

Strengthening by secondary phases depends on particle size, volume fraction, morphology, and spacing. Fine non-shearable particles impede dislocation motion through Orowan bypassing [38], whereas coarse particles and agglomerates strengthen less efficiently and can concentrate local stress.

*YS* ∝ *f* ^1/2^ *d*^−1^∙*lnd* (3)

where *YS* is the yield-strength increment arising from Orowan strengthening, *f* is the volume fraction of the secondary-phase particles, *d* is their mean diameter. A fine, uniform particle dispersion should therefore provide more effective strengthening than a coarsened or agglomerated distribution. The available data do not include the particle-size, volume-fraction, and interparticle-spacing measurements needed to calculate an Orowan increment.

The nonmonotonic tensile response arose from competition among these microstructural contributions. At 1 wt.% Al_2_Y, the mean KAM was highest, indicating the greatest retained deformation, while dispersed particles and texture supplied additional strengthening. This condition produced the maximum TYS and UTS but the lowest elongation. At 3 wt.%, the fine DRXed grains, bimodal structure, relatively uniform Al_2_Y and Al_2_Ca dispersions, and Al_8_Mn_5_ precipitates maintained high strength while allowing substantially more elongation. At 5 wt.%, particle agglomeration and microstructural coarsening reduced strength, whereas the larger DRXed fraction favored ductility. AXMW12083 consequently gave the best overall strength–elongation balance, although it was neither the strongest nor the most ductile composition.

### 4.2. Strength–Ductility Synergy and Fracture Behavior

**Figure 10:**
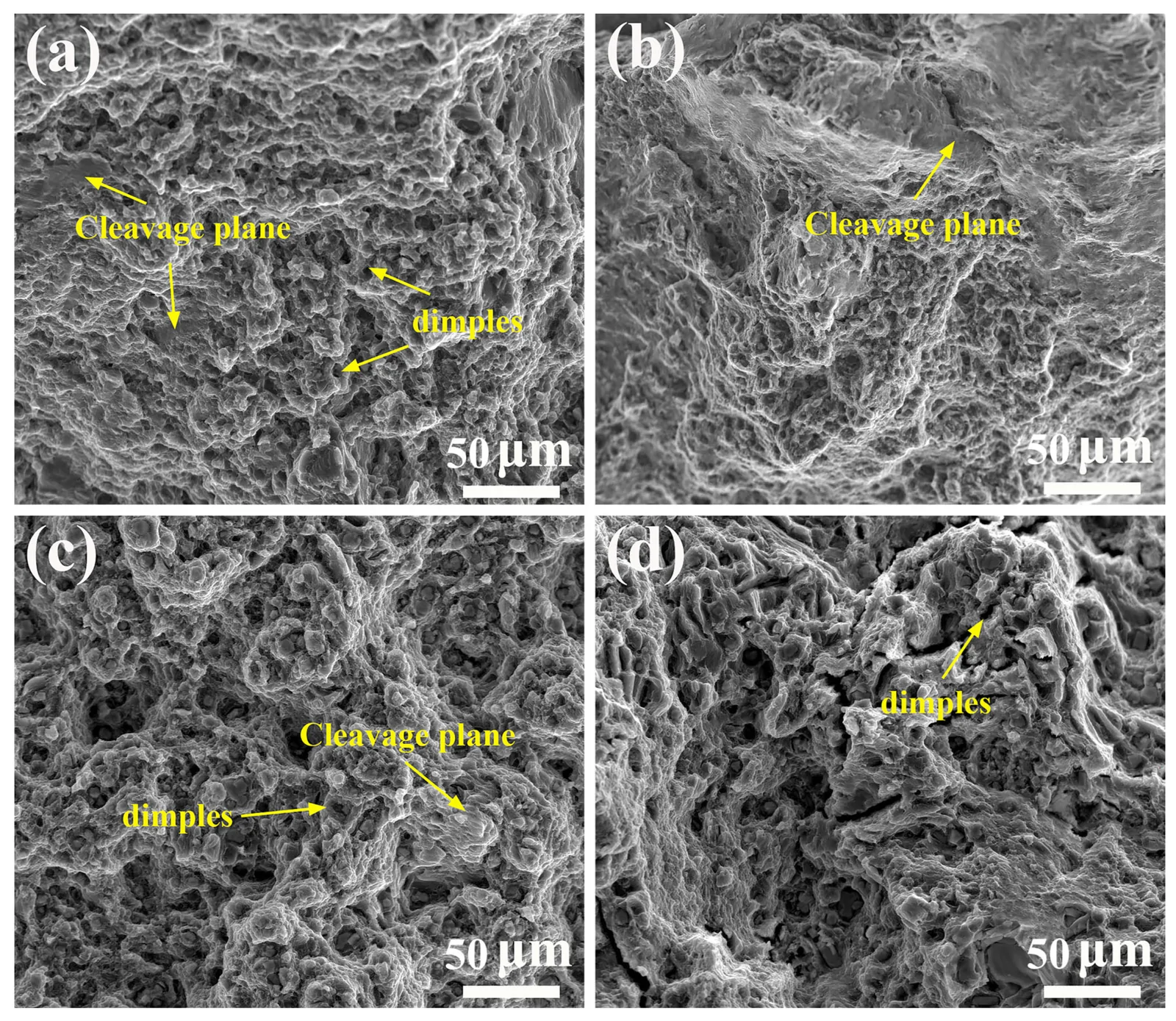
Fracture surfaces of the as-extruded AXMW1208X composites: (**a**) AXMW12080; (**b**) AXMW12081; (**c**) AXMW12083; (**d**) AXMW12085.

All fracture surfaces contained dimples, tear ridges, and quasi-cleavage facets (Figure 10), indicating mixed ductile and brittle fracture. AXMW12081 (Figure 10b) had a dimple morphology similar to that of AXMW12080 (Figure 10a). The dimples became larger and deeper at higher Al_2_Y contents, most clearly in AXMW12085 (Figure 10d). This change matches the tensile elongation: AXMW12085 was the most ductile composition, whereas AXMW12081 had the lowest elongation.

Al_2_Y particles may redistribute strain near the particle–matrix interface and thereby improve local deformation compatibility [39]. Crack deflection by the particles is also possible. Although both mechanisms would delay crack initiation or increase fracture-path tortuosity, the post-fracture surfaces alone do not establish the sequence of crack initiation and propagation.

The larger and deeper dimples in AXMW12083 and AXMW12085 are consistent with greater local plastic strain accommodation, whereas the quasi-cleavage facets indicate regions in which crack growth was less effectively blunted. Fragmented Al_2_Ca and Al_2_Y particles can act as local stress concentrators if debonding occurs, but they can also deflect a propagating crack when the particle–matrix interface remains sufficiently bonded. Because no interrupted tensile tests or in situ observations were performed, these interpretations remain mechanism-consistent rather than a direct reconstruction of the fracture sequence.

The fracture morphologies are thus compatible with the tensile results and with the proposed role of the heterogeneous microstructure. A moderate Al_2_Y addition improved the strength–elongation balance, whereas agglomeration at the highest addition reduced strength.

## 5. Conclusions

Mg–1.2Al–0.8Ca–0.4Mn composites containing 0, 1, 3, and 5 wt.% Al_2_Y were prepared by stir casting with a Mg–12Al_2_Y master alloy and then hot-extruded. The main findings are as follows:

(1) Master-alloy addition controlled the amount and distribution of Al_2_Y reinforcement. In the as-cast composites, Al_2_Y particles were present both within α-Mg grains and in interdendritic regions. The 3 wt.% Al_2_Y condition produced the finest structure, while 5 wt.% led to particle coarsening and agglomeration.

(2) Extrusion at 300 °C and a ratio of 7:1 produced fine DRXed grains alongside coarse unrecrystallized regions, while fragmented secondary phases aligned with the ED. Mean DRXed grain size decreased from 2.4 μm in AXMW12080 to 0.7 μm in AXMW12083. Rod-shaped Al_8_Mn_5_ particles in AXMW12083 may also contribute to dispersion strengthening.

(3) AXMW12081 had the highest strength (TYS 346 MPa; UTS 361 MPa) and an elongation of 5.8%. AXMW12083 combined a TYS of 316 MPa and a UTS of 332 MPa with 11.8% elongation, giving the best strength–elongation balance. At 5 wt.% Al_2_Y, elongation increased to 12.6%, but particle agglomeration and microstructural coarsening were accompanied by lower strength.

(4) The mechanical response depended on grain refinement, retained deformation, particle distribution, and extrusion texture. Under the processing and testing conditions used here, 3 wt.% Al_2_Y provided the most effective balance of particle dispersion, bimodal microstructure, strength, and elongation.

## Figures and Tables

**Table 1:** Nominal compositions of the AXMW1208X composites.

Designation	Al_2_Y Content (wt.%)	Matrix
AXMW12080	0	Mg–1.2Al–0.8Ca–0.4Mn (wt.%)
AXMW12081	1	
AXMW12083	3	
AXMW12085	5	

**Table 2:** EDS results of the as-cast composites shown in Figure 3 (at.%). Note: the D-point spectrum partially sampled the surrounding α-Mg matrix.

Position	Element				
	Mg	Al	Ca	Mn	Y
A	91.35	5.29	2.84	0.13	0
B	87.61	8.54	3.51	0.14	0
C	9.37	57.70	1.68	0	31.24
D	48.62	34.44	0	1.71	15.23

**Table 3:** Summary of tensile properties of the as-extruded composites at room temperature.

Designation	TYS (MPa)	UTS (MPa)	EL (%)
AXMW12080	285 ± 5	300 ± 9	6.5 ± 0.4
AXMW12081	346 ± 3	361 ± 8	5.8 ± 0.3
AXMW12083	316 ± 6	332 ± 6	11.8 ± 0.5
AXMW12085	296 ± 8	315 ± 8	12.6 ± 0.7

## Data Availability

The original contributions presented in this study are included in the article. Further inquiries can be directed to the corresponding authors.
